# Application of Microwaves to Reduce Checking in Low-Fat Biscuits: Impact on Sensory Characteristics and Energy Consumption

**DOI:** 10.3390/foods14152693

**Published:** 2025-07-30

**Authors:** Raquel Rodríguez, Xabier Murgui, Yolanda Rios, Eduardo Puértolas, Izaskun Pérez

**Affiliations:** AZTI, Food Research, Basque Research and Technology Alliance (BRTA), Parque Tecnológico de Bizkaia, Astondo Bidea, Edificio 609, 48160 Derio, Spain; rrodriguez@azti.es (R.R.); xmurgui@azti.es (X.M.); yrios@azti.es (Y.R.); iperez@azti.es (I.P.)

**Keywords:** biscuit breaking, mechanical properties, drying, cereal-based products, cookies, baking, energy cost, emerging processing

## Abstract

The use of microwaves (MWs) has been proposed as an energy-efficient method for reducing checking. Along with understanding moisture distribution, it is essential to consider structural characteristics to explain how MWs reduce checking. The influence of MWs on these characteristics depends on the food matrix’s dielectric and viscoelastic properties, which vary significantly between fresh and pre-baked dough. This study investigates the effects of MW treatment applied before (MW-O) or after conventional oven baking (O-MW) on low-fat biscuits that are prone to checking. Color (CIELab), thickness, moisture content and distribution, checking rate, texture, sensory properties, energy consumption and baking time were analyzed. The findings suggest that MWs reduce checking rate by eliminating internal moisture differences, while also changing structural properties, as evidenced by increased thickness and hardness. MW-O eliminated checking (control samples showed 100%) but negatively affected color, texture (increased hardness and breaking work), and sensory quality. The O-MW checking rate (3.41%) was slightly higher than in MW-O, probably due to the resulting different structural properties (less thickness, less hardness and breaking work). O-MW biscuits were the most preferred by consumers (54.76% ranked them first), with color and texture close to the control samples. MW-O reduced total energy consumption by 16.39% and baking time by 25.00%. For producers, these improvements could compensate for the lower biscuit quality. O-MW did not affect energy consumption but reduced baking time by 14.38%. The productivity improvement, along with the reduction in checking and the satisfactory sensory quality, indicates that O-MW could be beneficial for the bakery sector.

## 1. Introduction

Baking is crucial in biscuit production, affecting sensory and technological properties through processes such as water evaporation (moisture content below 6%), protein denaturation, starch gelatinization, dough expansion, and browning [1,2,3,4].

In an industrial context, baking is considered a low-efficiency and high-energy consumption process [3]. Traditionally, it is performed in forced convection ovens characterized by low heat transfer coefficients (approximately 30–300 W/m^2^ °C) [5]. Heat is transmitted to the food mainly by convection and conduction, although the effect of thermal radiation from the oven walls is not negligible [3]. In a world shaped by the climate crisis and the rising cost of resources, several innovative heating technologies have been proposed to improve the quality and efficiency of baking, including microwaves, radiofrequencies, infrared, halogen lamps, and, more recently, CO_2_ lasers [6,7,8,9,10]. Among these technologies, microwaves (MWs) have attracted significant interest [3,9,11,12]. Unlike forced convection ovens, which rely on convection, radiant and conduction heating, MWs’ effects are based on volumetric heating, thereby enhancing efficiency. MW energy is directly absorbed by certain molecules (dipoles such as water) based on their dielectric properties. These molecules rapidly switch polarity, generate friction, and produce heat that is transferred to nearby materials [6]. Additionally, MW baking has the potential to shorten baking time [6,7], thereby boosting production yield compared to conventional methods.

In addition to the high energy consumption of the baking process, producers of biscuits and other dry bakery products face unsolved challenges such as checking [4,12,13,14]. This phenomenon is defined as the occurrence of hairline cracks or fissures post-baking that extend partially across the central part of the biscuit [4,11]. These radial cracks can diminish the biscuit’s strength by up to 50% [15], rendering it more susceptible to breakage [4,15]. The factors that contribute to checking have been thoroughly studied. During conventional oven baking, substantial moisture differences typically occur between the outer (drier) and the inner parts (wetter) of the products [13,15]. During the cooling phase, as well as packaging and storage, the moisture content throughout the biscuit is equalized [15]. The sections of the biscuits that lose water shrink (center), while those that gain water expand (edges), which also take up moisture from the surrounding environment [16]. This water migration induces thermo-mechanical and/or hydro-mechanical stresses, potentially leading to checking and ultimately breakage [4,14]. While this is the primary mechanism linked to checking, factors related to composition (e.g., gluten, fat and sugar content) and processing (e.g., geometric shape, cooling rate), which impact the structure of the biscuit, also influence the formation of checking. Based on that, since the 1970s, various strategies have been applied to mitigate checking, both in formulation (e.g., reducing gluten, raising the pH above 7) and processing (e.g., slow cooling phase, hot packaging) [16]. Nonetheless, checking remains a prevalent problem, particularly in low-fat and low-sugar biscuits [13,17,18]. The diminished interaction of these components with gluten compromises matrix elasticity and its ability to absorb and dissipate post-baking internal stress, thereby increasing susceptibility to structural failure [16]. This issue impacts both consumers, through the presence of broken biscuits, and manufacturers, by causing considerable annual losses and substantial food waste, an area receiving growing attention from regulatory bodies and legislative authorities [19].

The use of MWs is noteworthy for the bakery sector not only for reducing baking time and energy consumption, but also for minimizing checking in biscuits and other dry products [3,11]. This is particularly pertinent when the product possesses attributes that facilitate checking, such as reduced fat content or a round shape [13,17,18]. The reduction in checking is primarily attributed to decreased internal moisture differences [11,20]. However, it is also essential to consider MW-mediated changes in food components (e.g., starch gelatinization, protein denaturation, water evaporation), product structure (e.g., expansion, crystallization, alveolation), and textural properties (e.g., increase in hardness, brittleness, crunchiness).

A reduction in checking will be futile if the process results in negative changes in the perceived appearance, odor, taste, and/or texture of the products, hindering their consumer acceptance. It is well known that MW-baked products often face issues like inadequate surface color, flavor, and texture [6,7,9,12]. To mitigate these issues, particularly color defects, the application of a conventional oven step either before (O-MW) or after MW treatment (MW-O) has been suggested [11,20].

While O-MW has been successfully tested in biscuits for checking reduction [11,20], MW-O has not received significant attention despite its potential. Applying microwaves to fresh dough with higher moisture content (e.g., 20% wb) can enhance baking efficiency. Higher moisture contents are associated with improved dielectric properties, specifically higher static and optical dielectric constants, in starch, gluten and related dough [21]. This phenomenon occurs due to the increased mobility of the mixture, as water acts as a plasticizer [21]. Based on that, MW-O may enable a notable reduction in both baking time and energy consumption compared to O-MW. The enhanced dielectric properties of the fresh dough may also facilitate the MW-mediated expansion of the biscuit structure. This would aid the subsequent removal of residual moisture during the conventional oven stage and limit internal moisture differences and checking. Since MW is applied to fresh dough with all its ingredients in their original state, it is anticipated that the properties of MW-O biscuits, such as thickness, color, texture, or sensory quality, will differ from those of conventional and O-MW biscuits.

The significance of energy consumption has increased in recent years due to escalating energy prices and the pressing issue of climate change. MW technology has been reported in general terms for its effectiveness in reducing energy consumption during baking processes [6,7]. However, the use of microwave (MW) technology in conjunction with a conventional oven may be less efficient. Additionally, energy consumption can vary significantly depending on whether MW is applied before or after using the conventional oven.

Based on these detected knowledge gaps, the aim of this work was to investigate the potential different effects of the application of MW before (MW-O) or after conventional baking (O-MW) on the sensory characteristics and checking rate of low-fat biscuits prone to checking. Studying the application of MW technology either before (on fresh dough) or after conventional oven baking (on pre-baked product) could yield valuable insights into how MW reduces checking in relation to moisture content distribution and structural properties, such as thickness and textural behavior. Finally, to advance the study of the potential industrialization of these MW processes, energy consumption and baking time, linked to productivity, were also compared and examined.

## 2. Materials and Methods

### 2.1. Dough Preparation

Biscuit dough was prepared using a recipe with reduced fat content [17,22] that induces a high checking rate to examine whether MW processes could mitigate checking issues under conditions that promote such defects. Other authors, aimed at reducing checking or analyzing certain parameters affecting it, used a similar approach [13,18]. The recipe consisted of wheat flour (62.50%; 11.7% protein content), white sugar (13.02%), water (11.88%), sunflower oil (10.05%), glucose (0.82%), sodium chloride (0.75%), ammonium bicarbonate (0.67%), and sodium bicarbonate (0.31%). Glucose was supplied by Chefdelice (Murcia, Spain), ammonium bicarbonate was supplied by Innovative Cooking (Madrid, Spain), and the rest of the ingredients were from the Makro Distribución Mayorista (Madrid, Spain).

All dry ingredients (wheat flour, white sugar, sodium chloride, ammonium bicarbonate and sodium bicarbonate) and sunflower oil were initially blended in a planetary mixer (BM-22, Sammic S.L., Azkoitia, Spain) using the spiral hook attachment at 46 rpm for 2 min. Glucose was previously diluted in the water and then the mixture was added and mixed at 46 rpm for 2 min. After manually scraping the dough from the sides of the bowl, the mixture was mixed again at 111 rpm for 5 min to achieve a homogeneous dough. Following a 30 min rest, the dough was sheeted with a pizza molder (FMI-31, Sammic S.L.) to a thickness of 3.1 ± 0.5 mm and cut with a circular die of 54 mm diameter. The round shape was chosen based on the findings of Bedas et al. [13], who found higher checking in round biscuits compared to rectangular ones. The raw biscuits (weight: 17.38 ± 0.23 g; moisture content: 21.22 ± 0.43% expressed on wet basis (wb)) were then immediately baked.

### 2.2. Baking Processes

Batches of 30 raw biscuits were baked following three different processes: forced convection oven (O), forced convection oven followed by microwave oven (O-MW), and microwave oven followed by forced convection oven (MW-O). The number of biscuits to be baked for each process, as well as their positioning, was optimized in previous experiments to obtain homogeneous biscuits in terms of color, humidity, and final weight. For the O process (control), biscuits were baked at 190 °C (preheated oven) for 16 min in a forced convection oven (WCAT41, De Dietrich, Rueil-Malmaison, France) with combined heat (fan, top, and bottom heat). These oven conditions are typical for this category of product [1,23,24]. O-MW baking started in the forced convection oven at 190 °C (preheated oven) for 12 min (fan, top, and bottom heat). After that, a treatment of 3.50 W/g of raw biscuits for 1.7 min was applied in an MW multimode oven Labotron FL8000 (8 KW, 2.45 GHz, 500 mm circular rotating platform) manufactured by Sairem (Lyon, France). The MW-O process involved baking the raw biscuits first in the described MW oven at 1.75 W/g for 6.5 min and finishing the process in the forced convection oven at 190 °C (preheated oven) for 5.5 min.

Baking conditions were selected after preliminary essays to obtain biscuits of standard quality in the shortest time, keeping final and intermediate moisture within the same range. This aspect has been established as crucial for valid comparisons between MW combined processes since moisture content affects checking, texture and other quality parameters of the biscuits [7,11,17], potentially masking the effects of MW on other food components related to these properties. Based on the MW experiments conducted by Ahmad et al. [11] and the Spanish regulation [2], a final moisture content of approximately 3% (2.69 ± 0.71% wb) was selected. In O-MW, an intermediate moisture content of around 8% (7.69 ± 0.25% wb) was defined since preliminary tests in the forced convection oven indicated that evaporation slowed considerably after reaching this threshold. Using MW at this low evaporation flux stage could speed up the process through volumetric heating. Previous studies have described this reduction in mass flux during conventional baking [23,24]. For instance, Dermirkol et al. [23] observed a decrease in evaporation flux at a moisture content of 7% wb (after baking for 14 min) in comparable biscuits (initial moisture content of 17% wb) and baking conditions (forced convection oven, 190 °C). The same intermediate moisture content was selected in MW-O to compare the use of microwave technology during phases typically defined by different water evaporation and product properties.

The MW power densities in O-MW (3.50 W/g; baking from a moisture content of 7.69 ± 0.25 to 2.69 ± 0.71% wb) and MW-O (1.75 W/g; baking from a moisture content of 21.22 ± 0.43 to 7.69 ± 0.25% wb) were selected as they were the highest values that allowed us to achieve the target moisture contents in the shortest time without overtreating the biscuits. Thus, in MW-O the use of power densities above 1.75 W/g led to noticeable and heterogeneous puffing (dome effect) and the formation of large air cavities inside the biscuits. In O-MW, using power densities beyond 3.5 W/g resulted in excessive internal roasting and even burning. These microwave power densities, and the resulting processing times, are within typical ranges for drying and baking foods with similar moisture contents [7,11,25].

Each baking process (O, MW-O, O-MW; batches of 30 biscuits) was repeated ten times, manufacturing a total of 300 biscuits per experimental condition. After cooling at room temperature (still air, 20 °C, 15 min), biscuits were individually packed in plastic containers (PE-PA, 120 µm; Vacioplast Salamanca, Salamanca, Spain) using a Multivac C200 machine (Multivac, Wolfertschwenden, Germany) and stored at room temperature (20 ± 2 °C) for up to 14 days before analysis. The study ended after 14 days as there were no changes in the checking rate, moisture content, or texture parameters compared to the 7-day results. Similar studies also concluded within this timeframe [11,14]. Samples (average weight of 13.80 ± 0.72 g, no significant differences between processes) were randomly grouped for each analysis.

### 2.3. Characterization of the Biscuits

#### 2.3.1. Color

The surface color characteristics, specifically lightness (L*), red–greenness (a*), and yellow–blueness (b*), were assessed using a colorimeter (model C-400, Konica Minolta Inc., Tokyo, Japan) with a D65 illuminant and a 10° standard observer angle. Measurements were conducted at the center of the biscuits (n = 6) for each baking process after 1 day of storage [10].

The total color difference (∆E) was determined using Equation (1):

∆E = [∆L*^2^ + ∆a*^2^ + ∆b*^2^]^0.5^ (1)

where ΔL*, Δa*, and Δb* correspond to differences between L*, a*, and b* values of O-MW and MW-O biscuits compared to the average control values (O samples). ΔE levels indicate how perceptible color differences are to the human eye [26]: ≤1 means “Not perceptible”, 1 < ΔE ≤ 2 is “Perceptible through close observation”, 2 < ΔE ≤ 3 is “Perceptible at a glance”, ΔE > 3 is “Noticeable difference”, ΔE > 10 means that “Colors are more similar than opposite”, and ΔE > 50 indicates “Clearly noticeable difference”.

The Browning Index (BI) of the samples [3] was determined using Equation (2):

BI = 100 × (x − 0.31)/0.172(2)

where x was calculated following Equation (3):

x = (a* + 1.79L*)/(5.645L* + a* − 3.012b*)(3)

#### 2.3.2. Thickness

The thickness (mm) was measured after 1 day of baking in sextuples (n = 6) using a manual caliper (Mitutoyo, Kawasaki, Japan). Measurements were taken at the center (inner thickness) and 10 mm from the edge (outer thickness).

#### 2.3.3. Checking Rate

The occurrence of checking, defined as the manifestation of hairline cracks on the surface of the biscuits, was assessed through visual inspection [11]. Two batches of 75 biscuits (a total of 150 biscuits) were monitored for each baking process (O, O-MW, MW-O). The checking rate (%) was calculated by counting the number of biscuits exhibiting checking after 1, 7, and 14 days of storage post-baking.

#### 2.3.4. Moisture Content

The moisture content (MC, % wb) of biscuits (10 samples per baking process) was measured on days 1 and 14 by drying at 105 ± 1.5 °C until constant weight was reached (Memmert UFP800/B0, Genesys Instrumentación, Madrid, Spain). MC was analyzed in the central (Cen), middle (Mid), and external (Ext) parts of the biscuit, using dies of 42 and 21 mm diameter to cut the sample.

#### 2.3.5. Texture

The instrumental texture was measured using an HD Plus Texture Analyzer (Stable Micro Systems Ltd., Surrey, Godalming, UK) equipped with a 30 kg load cell. The measurements (n = 24) were performed after 1 day of storage following a method adapted from Talens et al. [27]. The biscuits were placed on the heavy-duty platform (HDP) and holed plate and then penetrated in the center to a depth of 10 mm using a flat-ended 4 mm aluminum cylindrical probe (SMP/4) at a speed of 40 mm/s (pre-test speed of 1 mm/s, post-test speed of 10 mm/s), and a trigger force of 5 g. The results were analyzed with the Exponent 6.1.17.0 software (Stable Micro Systems Ltd.). The parameters obtained from the force–time curves were the peak force needed to break the biscuit (hardness, g) and the area under the curve up to this peak force (breaking work, g s).

#### 2.3.6. Sensory Analysis

The sensory analysis was conducted after 1 day of storage. A trained panel (n = 7) assessed the sensory profile, while consumers (n = 42; 23 to 59 years, 59% female) evaluated acceptability and preference. The study did not need to be reviewed and approved by the Medical Research Ethical Committee of The Basque Country, in accordance with the “Medical Research Involving Human Subjects Act” of The Basque Country. In any case, the study complied with the Helsinki Declaration [28], and informed consent was obtained from each participant. Tests were conducted under white light in a sensory laboratory. The analysis took place the day after baking, with the biscuits at room temperature (20 ± 2 °C). Each panelist tasted three biscuits (one per baking condition) presented simultaneously on white porcelain dishes coded with random three-digit numbers.

The trained panel underwent training sessions in accordance with ISO 8586:2023 [29], using commercial biscuits and custom patterns. During these training sessions, product descriptors were created, and rating scales were established and learned. The sensory characteristics of the biscuits were evaluated using Quantitative Descriptive Analysis (QDA), applying a structured 11-point scale [30], from 0 (non-perceptible) to 10 (very intense). The descriptors assessed (Table 1) included appearance (external color), texture (toughness, fragility, granularity, and mouth residue), flavor (typical, non-typical, and sweetness), and odor (typical and non-typical).

Consumer acceptability of biscuits was evaluated based on odor, appearance, flavor, texture, and overall impression using a structured 9-point scale [31], ranging from 1 (dislike extremely) to 5 (neither like nor dislike) to 9 (like extremely). A sorting test [32] was conducted to assess preferences among biscuits baked under the three different conditions tested.

### 2.4. Energy Consumption and Baking Time

Energy consumption (Wh) for each baking condition (O, O-MW and MW-O) was determined using a power meter (PW3337, Hioki E.E. Corporation, Ueda, Japan) in both oven and MW phases (n = 5). Subsequently, the energy consumption per kilogram of raw biscuits (EC, Wh/kg) of each phase and baking condition was calculated. The total baking time (min) was determined as the sum of the duration of each individual phase.

### 2.5. Statistical Analysis

Unless otherwise specified, the results were expressed as mean ± 95% confidence interval (95 CI). The statistical analyses were conducted using the XLSTAT v. 2105 software (Lumivero, Denver, CO, USA). One-way ANOVA was performed for thickness, color, instrumental texture, and sensory analysis (trained panel, QDA; consumer panel, acceptability test), while two-way ANOVA was conducted for checking rate and moisture content. In all cases, the analysis of variance was followed by Tukey tests (*p* = 0.05). Chi-square test (*p* = 0.05) was applied to frequency of choice data for the preferred biscuits (consumer panel, sorting test).

## 3. Results and Discussion

### 3.1. Color

Regardless of the baking process, no statistical differences (*p* ≥ 0.05) were observed in L*, a*, and b* parameters measured in the central zone of the biscuit surface 1 day after baking (Table 2). The values obtained fall within the usual range for this type of product [7,18]. Therefore, when MW conditions are properly optimized, its application before (MW-O) or after a conventional oven process (O-MW) results in biscuits with acceptable surface color.

Browning, based on Maillard reaction, caramelization, and dextrinization, is primarily responsible for the characteristic color of cereal-based baked products [3]. It is well known that MW slightly produces browning on the surface of these foods [6,10]. Achieving the necessary browning requires combining MW with conventional ovens or other technologies like infrared or halogen lamps [7,10]. The Browning Index (BI), calculated from L*, a*, and b* parameters [3], showed no significant differences (*p* ≥ 0.05) between the baking methods (O, O-MW, MW-O), indicating appropriate conditions. However, noticeable variations in ΔE (values between 3 and 10) were found between MW-based treatments and the control method (O). Nevertheless, no significant differences (*p* ≥ 0.05) were detected between the two treatments that include an MW step.

### 3.2. Thickness

The raw biscuits had an initial thickness of 3.1 ± 0.5 mm, with no significant difference between inner and outer zones (*p* ≥ 0.05). During baking, this dimensional variable increases due to water vaporization, gas expansion, and CO_2_ production by the leavening agents, depending not only on the recipe but also on the baking conditions [3,18,20]. O-MW and MW-O biscuits showed significantly larger inner thickness than O controls (*p* < 0.05) (Table 3). Although the average outer thickness values were slightly lower than the inner thickness ones for the three types of biscuits, no significant differences were found (*p* ≥ 0.05). Bernussi et al. [20] also reported greater thickness in biscuits baked by combining conventional and microwave ovens (O-MW), although the differences were smaller. This could be explained by the different recipes and MW treatments applied.

Unlike conventional heating systems, microwaves heat the entire sample (volumetric heating), facilitating moisture migration and product expansion. The rapid rise in internal temperature can cause localized superheating, where water remains in a liquid state above its boiling point due to pressure constraints. When nucleation sites (such as entrapped air pockets or starch granules) are activated, this superheated water undergoes an abrupt phase transition, generating vapor bubbles and vapor flux. This imposes mechanical stress on the viscoelastic matrix, causing the product volume to expand [33]. The rate of vapor release depends on internal pressure gradients, pore connectivity, and matrix permeability. The dough fluidity governs the matrix’s ability to deform, accommodate expanding steam bubbles and canalize the vapor outflow.

Applying the microwaves before the conventional oven (MW-O) resulted in higher inner thickness (*p* < 0.05) than applying them afterward (O-MW). The disparity in expansion could be attributed to the variations in vapor flux and product characteristics when applying MW in both alternative processes. During the MW phase in MW-O, the product (fresh dough) presents higher initial moisture content and, consequently, better dielectric properties [21] than in O-MW (pre-baked product), generating higher volumetric heating, vapor flux and expansion. Furthermore, in the MW phase in MW-O the fresh dough has not yet achieved rigid structural setting through starch gelatinization or protein denaturation, promoting higher matrix deformation and expansion.

### 3.3. Checking Rate

Checking was evaluated for 14 days after baking, which is considered enough time for the issue to appear [11,20]. In conditions that promote the presence of moisture differences among biscuit parts, checking can appear even within the first 24 h of storage [13]. As expected from the literature [11], checking (hairline fissures) appeared primarily in the central region of the top surface and occasionally on the bottom surface of the biscuit.

Conventional baked biscuits (O) had a checking rate of 94.74 ± 8.07% after 1 day of storage, increasing to 100% after 7 days (Table 4). Producing these biscuits industrially would require substantial modifications to the recipe and/or the process to achieve a reasonable checking rate. Building on the work of Ahmad et al. [11] and Bernussi et al. [20], MW treatment before or after conventional baking could reduce checking rate in these naturally prone-to-checking biscuits without having to alter the formula or the rest of the process variables. Other authors who investigated the effects of certain parameters on checking, like moisture distribution, biscuit shape or the use of sweeteners, also used a control formula that favored checking [13,18]. For instance, Roze et al. [18] reported high checking rates (93–95%) after 72 h of storage in relative low-fat biscuits (13.3%).

Microwave treatment before (MW-O) or after (O-MW) the conventional oven process dramatically reduced the checking rate in biscuits (*p* < 0.05). This decrease would be mainly explained by the elimination of moisture differences between the biscuit’s parts (Table 5), the main cause of checking according to bibliography [14,15]. This is explained and discussed in more depth in the section on moisture content (Section 3.4). Unlike the control, no new biscuits showed checking problems after 1 day of storage, demonstrating microwaves’ effectiveness in controlling this unsolved issue. During the 14-day study, O-MW biscuits showed a checking rate of 3.41 ± 2.99%. This value is similar to those previously reported for equivalent processes [11,20]. For example, Ahmad et al. [11] reported a 5% average checking rate when baking analogous biscuits in a reel oven (245 °C; 3 min) followed by the application of MW (700 W, 30 s).

**Figure 1:**
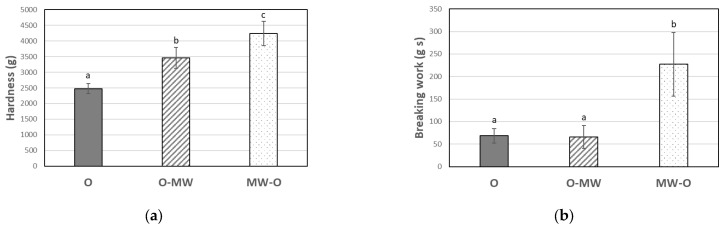
Biscuit hardness (g) (**a**) and breaking work (g s) (**b**) as a function of baking method, O (solid), O-MW (scratched) and MW-O (pointed), after one day of storage. Means ± 95 CI (n = 24). Different letters denote significant differences for each parameter (*p* < 0.05) based on the baking process.

MW-O baking not only significantly reduced checking compared to O or O-MW (*p* < 0.05) but also eliminated it entirely, as none of the 150 biscuits showed fissures. Considering that MW-O and O-MW exhibited balanced internal moisture content without significant variances over the 14-day evaluation period (Table 5), the differences in checking rates between these processes may be attributed to other properties. The application of microwave treatment to fresh (MW-O) or pre-baked biscuits (O-MW), which are distinguished by different initial moisture contents, dielectric properties, levels of viscoelasticity, and structural characteristics, resulted in biscuits with distinct final structural and textural features. MW-O biscuits presented statistically higher inner thickness (Table 3) as well as higher hardness and breaking work values than O-MW (Figure 1). This more expanded and rigid structure compared to O-MW could prevent checking (Section 3.5). The O-MW biscuits also showed greater expansion and hardness than the control biscuits (Table 3 and Figure 1). Further research is necessary to gain a comprehensive understanding of the mechanisms underlying MW-mediated reductions in checking and their relative significance in this phenomenon.

### 3.4. Moisture Content

Differences in moisture content inside the biscuits is one of the main parameters that influence the occurrence of checking [14,15,18,22]. To examine the effect of the MW combined processes on moisture distribution and its relation with checking rate, the moisture content (MC, % wb) was measured in the central (MC Cen), middle (MC Mid), and external (MC Ext) parts of the biscuit, as well as in the whole sample (MC total), after 1 and 14 days of storage (Table 5).

In a conventional forced convection oven, the biscuit is heated from the outside inwards. Consequently, mass and heat transfer occur in opposite directions, leading to noticeable differences in MC between the inner and outer parts of the biscuit [13,15]. During the cooling phase, packaging, or storage, water migrates to equalize MC, inducing internal thermo-mechanical and/or hydro-mechanical stresses, potentially leading to checking and ultimately breakage [4,15,17]. After 1 day the conventionally baked samples (O) showed significant variations in MC depending on the biscuit zone (*p* < 0.05). The moisture difference between the center and the middle of the O samples was 1.53% wb and 1.52% wb between the middle and external parts, totaling a 3.05% wb difference between the center and outside. These important moisture differences would be directly related to the 94.74% checking rate of O biscuits after just one day (Table 4). The biscuits normally checked along the diameter, where stresses were highest due to the radial moisture differences, rather than at the edge. Shallow, short, radial cracks near the center are more damaging than deeper and longer cracks near the biscuit circumference [15].

MW treatment ensured a homogeneous moisture distribution (*p* ≥ 0.05) whether applied before (MW-O) or after the conventional oven process (O-MW), with moisture differences between biscuits parts below 1% wb. These results agree with Bernussi et al. [20], who observed similar moisture differences when using MW heating (0.5 min at medium power, 82.8% of 800 W) after conventional baking (240 °C, 4 min). As MW technology is a volumetric heating method [6], both mass and heat transfer occur in the same direction, from the interior towards the exterior of the biscuit. This heating mechanism creates a driving force for moisture flow and produces higher vapor pressure inside than at the surface, which promotes a consistent mass flux from the inner to the outer regions, facilitating evaporation and reducing internal moisture differences. This homogeneous moisture distribution significantly reduces internal tension forces, minimizing the occurrence of checking [11]. The low checking rates of O-MW and MW-O samples (Table 4) confirm this hypothesis.

During storage, the MC of baked products balances internally, depending on the diffusion of water between their different areas, and externally, between the products and the surrounding environment [11,13]. After 14 days, MC increased across all baking processes (Table 5). Although biscuits were individually packaged in impermeable plastic (PE-PA), they were not vacuum-packed to avoid texture alterations and their potential impact on checking. Results suggest that some water from the internal environment was absorbed by the biscuits. As the checking rate remained stable for 14 days (Table 4), this moisture gain’s impact is considered negligible.

Significant differences in moisture content were observed between the central (MC Cen) and external parts (MC Ext) of O samples after 14 days (*p* < 0.05). All O biscuits already had a 100% checking rate by day 7 (Table 4), so these moisture differences had no practical implications. Meanwhile, MC remained similar across all parts of O-MW and MW-O biscuits after 14 days (*p* ≥ 0.05). This may be a primary reason for the lack of checking during storage in these samples.

### 3.5. Texture

The baking method (O, O-MW, and MW-O) statistically affected the hardness (g) and breaking work (g s) (Figure 1). Texturometric properties are closely linked to checking and breakage behavior [15]. Moisture content is a critical characteristic that defines the textural properties of baked products [10]. In earlier studies on the use of MW in biscuits, each baking condition resulted in different final moisture levels, which could mask the effects of this technology on the texture [7,20]. In the present experiment, its influence was ruled out as similar total MC was found among the different samples (Table 5).

O-MW biscuits showed higher peak force to break (hardness) than O ones (*p* < 0.05), with MW-O samples being even harder (*p* < 0.05). MW-O samples also required three times more breaking work (*p* < 0.05) compared to O and O-MW, which showed no significant differences between them (*p* ≥ 0.05). It is well known that MW can provoke increased crumb firmness and rapid staling in baked goods [6,9,12,34]. Low moisture differences between the different parts of the biscuit, like the ones found in O-MW and MW-O samples, are linked to high hardness and low checking rates [7,11,15,18]. However, the large textural differences in this research cannot be solely explained by this cause. In addition, although O-MW and MW-O processes showed no internal moisture differences (Table 5), they displayed differences in checking rate (Table 4) and significant texture variances (Figure 1).

In MW-O, microwaves were used on fresh dough with high initial MC and ingredients in their native form, whereas in O-MW, microwaves were applied to a pre-baked product that had lower initial MC and had undergone some starch gelatinization or protein denaturation. Consequently, in both baking methods, the products exposed to microwave treatment exhibited distinct initial dielectric, viscoelastic, and structural characteristics. MWs modify food components (e.g., starch, gluten) and vapor flux, leading to increased expansion and other structural changes [9,25,33,35]. These changes depend on the properties of the product and the MW treatment applied. The complex interaction of all these factors could explain the textural contrasts and the checking rate differences between the O-MW and MW-O samples.

MW treatments (<5 W/g) convert native starch crystallinity from B to A in relatively low-moisture models (<30% wb) [35]. For example, Luo et al. [36] observed this phenomenon applying MW in maize (1 W/g for 20 min; MC: 30% wb), whereas no such change was noted with conventional heating. Recently, Song et al. [37] examined type A and B starch gels from three wheat varieties, finding that type A gels were harder than type B ones. This may partly explain why MW-O biscuits were harder than O-MW samples. In MW-O, microwaves were applied to raw starch (fresh dough), shifting its crystallinity from type B to A, whereas in O-MW, the starch was already pregelatinized (B type) by prior oven treatment. An increase in the firmness of MW-baked biscuits would be a result not only of starch changes. MW heating can align gluten proteins through strong hydrogen bonds, which leads to greater firmness during cooling [34]. This could be especially relevant in the present case, as the protein content of the flour used was relatively high (11.7%).

Furthermore, MW evaporates water within the whole treated mass (volumetric heating), expanding the product [9,33]. During the vapor release, it can carry away compounds that are deposited on the outermost part of the food, which could be related to an increase in hardness and a reduction in the occurrence of superficial fissures related to checking. As mentioned above, MW-O caused higher hardness and breaking work than O-MW (*p* < 0.05). MW-O baking applied microwaves to raw biscuits with high MC (21.22 ± 0.43% wb), resulting in significant water evaporation (13.53% wb) compared to O-MW (5.32% wb), which used pre-baked biscuits with lower moisture content (7.69 ± 0.25% wb). Thus, MW evaporated more water in MW-O than in O-MW, which would possibly drag a larger number of compounds in its outflow. Finally, as MW was applied in MW-O to crude dough (higher initial MC; original dielectric and viscoelastic properties), this led to more expansion than in O-MW (Table 2). Chang et al. [33] studied the texture of cassava cuttlefish crackers expanded by MW. These authors found that crackers with greater expansion and thickness exhibited higher breaking energy.

The presented results highlight that MW can reduce the checking rate not only by eliminating the moisture differences between biscuit parts but also by altering the structure and textural properties of the food. These findings emphasize how structural properties like thickness and the expansion grade or the differences in water evaporation patterns and MW-mediated changes in starch during baking significantly influence textural attributes and other important characteristics in baked goods. Further research is needed to understand how MW impacts the structure formation of cereal-based baked products, especially those pre-baked by other methods. This would allow a better comprehension of the process for better modulation and definition of microwave conditions to optimize baking results.

### 3.6. Sensory Analysis

Although the use of MW after conventional baking has been previously explored by other authors to reduce checking in biscuits [11,20], there is no published data about their effects on sensory properties, a crucial aspect for industrial application.

Quantitative Descriptive Analysis (QDA) indicates that microwaving before (MW-O) or after conventional oven use (O-MW) results in significantly less intense color (*p* < 0.05) compared to control baking (Table 6). This is supported by relatively high ΔE values, which pointed out noticeable variations in color for the human eye between microwaved and control samples (Table 2). Regardless of baking conditions, biscuits showed similar values (*p* ≥ 0.05) in fragility, granularity, mouth residue, typical and non-typical flavor, sweetness, and typical and non-typical odor. Other authors also found no difference in odor and flavor attributes between microwaved and conventional baked products such as muffins [9]. O and O-MW biscuits exhibited similar toughness (*p* ≥ 0.05). Conversely, MW-O samples showed higher values in this attribute (*p* < 0.05). Instrumental textural analysis revealed that both O-MW and MW-O biscuits had higher hardness values than O controls, while breaking work was significantly higher only in MW-O samples (Figure 1). Although these instrumental and sensory parameters are not directly comparable, it appears that the small but statistically significant differences in instrumental hardness between O and O-MW samples were not perceived in the toughness evaluation by trained panelists.

**Figure 2:**
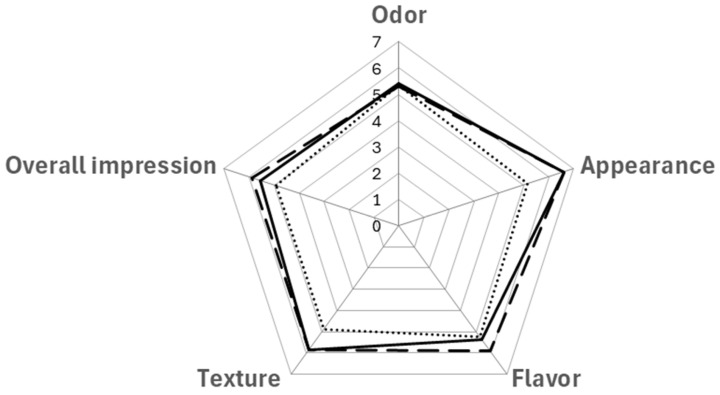
Values for odor, appearance, flavor, texture and overall impression of the biscuits based on the baking method, O (solid line), O-MW (dashed line) and MW-O (dotted line), as evaluated by the consumer panel (n = 42).

**Figure 3:**
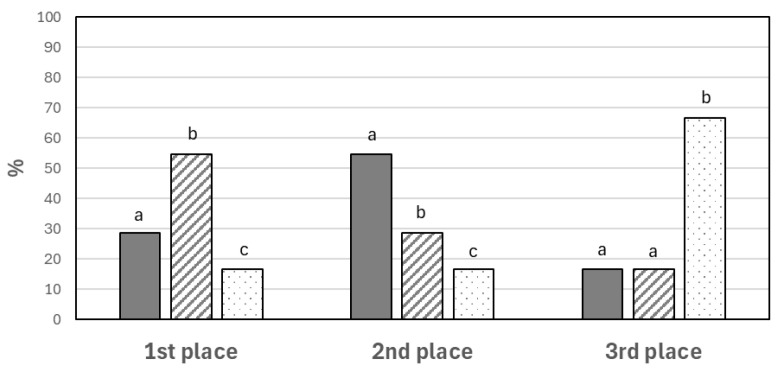
Ranking of the biscuits (sorting test) based on the baking method, O (solid), O-MW (scratched) and MW-O (pointed), according to the consumer panel (n = 42). Means ± 95 CI. Different letters denote significant differences between samples (*p* = 0.05).

Consumers did not notice any significant differences between O and O-MW processes (*p* ≥ 0.05) in any of the attributes evaluated (odor, appearance, flavor, texture, and overall impression) (Figure 2). However, O-MW biscuits scored higher in flavor and overall impression, with 54.76% of consumers choosing them as the most liked (*p* < 0.05) (Figure 3). The changes in the color of the O-MW biscuits observed by the trained panel (Table 6), as well as in ΔE value (Table 2), would not reflect negatively on the appearance, overall impression, and preference according to the consumers.

The application of MW before conventional oven use (MW-O) led to divergent results in certain attributes of the biscuits, with 66.67% of consumers rating them as the least liked (*p* < 0.05) (Figure 3). While O and O-MW samples scored 6.6 in appearance, the biscuits baked using MW-O were rated lower (*p* < 0.05), reaching a score of 5.1 (Figure 2). This would be due to the differences in color (Table 2) and greater expansion (higher inner thickness, Table 3) of the biscuits, which was not appreciated favorably by consumers. The application of MW-O resulted in significantly lower texture scores, consistent with the highest hardness and breaking work recorded (Figure 1). Additionally, the overall impression was lower (but acceptable) compared to O and O-MW. However, significant differences (*p* < 0.05) in overall impression were only observed between the MW-O and O-MW samples. This would be in line with the preference study, as they are respectively the least and most preferred biscuits by consumers (Figure 3).

In summary, MW-O samples were rated the lowest and were the least preferred. Significant alterations in color and texture were observed in the MW-O-baked products by both trained panelists and consumers. While the trained panel identified a less intense color in the O-MW biscuits compared to the O biscuits, this difference was not perceived by the consumers. Notably, consumers awarded the highest ratings for flavor and overall impression to the O-MW biscuits, making them the most preferred.

### 3.7. Energy Consumption and Baking Time

MW is considered an efficient heating technology since it is based on volumetric heating instead of the convection and conduction of conventional heating [6]. Based on this principle, a reduction in both total EC and total baking time was expected (improved heating speed, less heat loss). Considering the preheating phase of the oven treatments, MW reduced the total baking time by 9.38% (O-MW) or 16.33% (MW-O) compared to the control O process of 24.5 min (Table 7). As expected, the MW-O process resulted in a significant reduction in total EC by 10.76% (*p* < 0.05). However, there was no significant difference in total EC between O and O-MW (*p* ≥ 0.05). In MW-O, the MW EC constituted 36.21% of the total value. In contrast, in O-MW, the MW EC represented only 16.71%.

In industrial baking, the impact of energy consumption related to preheating the oven is minimized as it is only carried out at the beginning of production (normally once a day), and then the oven operates for extended periods (hours) and several baking cycles are performed continuously. In order to avoid its influence and get closer to the reality of industrial production, the results in Table 7 are also shown excluding the EC of preheating the oven (285.7 ± 6.1 Wh/kg). In view of this, MW-O presented significantly lower total EC (456.4 ± 5.9 Wh/kg) than the O process (*p* < 0.05). Thus, once the oven is preheated and prepared for production, the potential reduction in energy consumption of MW-O would be 16.39%. Furthermore, MW-O reduced the total baking time by 25.00% (12 min) compared to the control process (16 min). Based on these figures, in an 8 h shift of MW-O could increase production by 33.33%. However, MW-O produces negative effects on the biscuits (noticeable sensory changes, increased expansion, greater instrumental hardness and breaking work). It is up to the food processors to determine whether the potential energy, economic, and processing time savings, coupled with the reduction in checking problems, would compensate for the lower quality of the products.

Excluding the EC of oven preheating, the O-MW process showed a total EC of 549.0 ± 5.1 Wh/kg, with no significant difference (*p* ≥ 0.05) compared to the O process (545.9 ± 2.9 Wh/kg). It seems that the improved heating efficiency resulting from the use of microwaves (volumetric heating) would not compensate for the relatively lower rate of conversion of electrical energy into microwave energy (70%). Although O-MW did not reduce energy consumption, it reduced the total baking time by 14.38% (13.7 min) compared to the control process (16 min). In an 8 h shift, O-MW could lead to a production increase of 16.79%. This estimated productivity gain, coupled with the drop in checking and breakage problems without major impacts on product quality and energy consumption, seems to indicate that O-MW could be a very interesting process for the bakery sector.

Although the energy and processing time results presented in this work are promising, additional studies are required to obtain figures at pilot and industrial scales that allow us to determine the feasibility and cost-effectiveness of using either O-MW or MW-O.

## 4. Conclusions

MW can strongly reduce checking rates even if biscuit formulation and characteristics promote it (low fat content, round-shaped). This is of great industrial relevance. MW technology has the potential to help manufacturers to produce biscuits that would be difficult or even impossible to produce without making variations on the formula and/or the process to achieve a standard checking rate.

The application of MW after (O-MW) or before conventional pre-baking (MW-O) significantly decreased the checking rate after 14 days of storage, from 100% (O samples) to 3.41% and 0%, respectively. This reduction would mainly be explained by the fact that MW eliminated the moisture differences between the different parts of the biscuit. However, the differences in checking rate between MW-O and O-MW suggest that there could be other MW effects playing a role. These MW effects would be determined by the particular dielectric, viscoelastic and structural properties of the food matrix (fresh dough or pre-baked biscuit) at the time of MW application.

MW-O biscuits presented higher thickness, hardness and breaking work than O-MW ones. This more expanded and firm structure could be linked with the lower checking rate found in MW-O biscuits. Further research is necessary to gain a comprehensive understanding of the mechanisms underlying MW-mediated reductions in checking and their relative significance.

MW-O resulted in significant and negative variations in product quality (ΔE, instrumental hardness and breaking work) compared to both O and O-MW processes. These important differences were perceived in the sensory tests by both the trained panel and the consumers, which could call into question MW-O’s applicability. On the contrary, changes detected in the O-MW biscuits were not detected as negative in the sensory analysis. In fact, over half of consumers preferred the O-MW biscuits over the O biscuits.

Scientific literature reports that MW reduces energy consumption in baking processes. However, the results of the present work point out that the application of MW in sequential combination with conventional ovens may not be as efficient. Excluding oven preheating, while O-MW did not decrease energy consumption, MW-O presented total energy consumption and baking time that were lower than in the O process. Food processors must evaluate whether the potential benefits of MW-O in terms of energy, economic savings, and processing time, along with the decrease in checking issues, justify the lower quality of the products. Although O-MW did not decrease energy consumption, it reduced the total baking time compared to the control process. The potential productivity gain associated with this shorter baking time, along with the reduction in checking and breakage issues without significantly affecting product quality and energy consumption, suggests that O-MW could be a beneficial process for companies producing biscuits and other baked goods affected by checking.

Additional research is required to elucidate the impact of variables such as fat, sugar, and initial moisture content on the effects of O-MW and MW-O processes on biscuit quality, as well as to assess their applicability to other baked products. The combination of MW with other energy sources in a single step, such as convective (e.g., forced air, air impingement), radiant (e.g., infrared, halogen lamp) or conductive heating (e.g., susceptors), rather than using sequential baking, may contribute to reducing process time and increasing efficiency.

## Figures and Tables

**Table 1:** List of descriptors and definitions used in the Quantitative Descriptive Analysis (QDA) by the trained panel.

Descriptors	Definitions
Color	External color of the biscuit (0 (white) to 10 (brown)).
Toughness	Force required to compress and break the biscuit between the teeth.
Fragility	Ability to break the biscuit during chewing into small pieces.
Granularity	Size and quantity of granules.
Mouth residue	Quantity of residue that remains in the mouth after swallowing.
Typical flavor	Perception of typical biscuit flavor.
Non-typical flavor	Perception of non-typical flavors.
Sweetness	Perception of sugar taste.
Typical odor	Perception of typical biscuit odor.
Non-typical odor	Perception of non-typical odor.

**Table 2:** Biscuit color parameters in the central zone of the biscuit surface based on the baking method (1 day of storage).

	L*	a*	b*	BI	ΔE
O	62.51 ± 2.68 ^A^	8.38 ± 0.80 ^A^	32.42 ± 1.30 ^A^	79.98 ± 5.94 ^A^	-
O-MW	64.80 ± 3.42 ^A^	7.41 ± 1.14 ^A^	33.07 ± 1.27 ^A^	76.92 ± 7.12 ^A^	4.72 ± 1.67 ^A^
MW-O	59.40 ± 4.94 ^A^	6.70 ± 1.47 ^A^	31.75 ± 2.70 ^A^	80.01 ± 7.60 ^A^	6.96 ± 3.11 ^A^

All values are means ± 95 CI (n = 6). Different capital letters in the same column indicate significant differences (*p* < 0.05) based on the baking process.

**Table 3:** Inner and outer thickness (mm) of the biscuits baked in a forced convection oven (O) or by the application of a microwave treatment after (O-MW) or before (MW-O) the oven.

	Inner Thickness (mm)	Outer Thickness (mm)
O	5.2 ± 0.2 ^aA^	4.9 ± 0.4 ^aA^
O-MW	5.8 ± 0.2 ^aB^	5.3 ± 0.5 ^aA^
MW-O	6.3 ± 0.3 ^aC^	5.7 ± 0.6 ^aA^

All values are means ± 95 CI (n = 6). Different lowercase letters in the same row indicate significant differences (*p* < 0.05) based on the biscuit zone. Different capital letters in the same column indicate significant differences (*p* < 0.05) based on the baking process.

**Table 4:** Checking rate (%) of the biscuits (O, O-MW and MW-O) at 1, 7 and 14 days after baking.

	Checking Rate (%) (Day 1)	Checking Rate (%) (Day 7)	Checking Rate (%) (Day 14)
O	94.74 ± 8.07 ^aA^	100.00 ± 0.00 ^bA^	100.00 ± 0.00 ^bA^
O-MW	3.41 ± 2.99 ^aB^	3.41 ± 2.99 ^aB^	3.41 ± 2.99 ^aB^
MW-O	0.00 ± 0.00 ^aC^	0.00 ± 0.00 ^aC^	0.00 ± 0.00 ^aC^

All values are means ± 95 CI (2 batches, 75 samples per batch). Different lowercase letters in the same row indicate significant differences (*p* < 0.05) based on the day. Different capital letters in the same column indicate significant differences (*p* < 0.05) based on the baking process.

**Table 5:** Moisture content (MC, % wb) of the biscuits at 1 and 14 days after baking, based on the applied process (O, O-MW, MW-O) and the part of the biscuit (central—Cen; middle—Mid; exterior—Ext; total).

	MC Cen (%)	MC Mid (%)	MC Ext (%)	MC Total (%)
**Day 1**				
O	4.88 ± 0.57 ^aA^	3.35 ± 0.47 ^bA^	1.84 ± 0.33 ^cA^	2.88 ± 0.36 ^bA^
O-MW	2.17 ± 0.78 ^aB^	2.12 ± 0.79 ^aA^	2.65 ± 0.47 ^aA^	2.37 ± 0.62 ^aA^
MW-O	2.90 ± 0.32 ^aB^	2.66 ± 0.23 ^aA^	2.93 ± 0.55 ^aA^	2.82 ± 0.31 ^aA^
**Day 14**				
O	4.39 ± 0.53 ^aA^	4.13 ± 0.38 ^abA^	3.30 ± 0.45 ^bA^	3.73 ± 0.41 ^abA^
O-MW	3.76 ± 1.16 ^aA^	3.46 ± 1.23 ^aA^	3.89 ± 1.02 ^aA^	3.70 ± 1.08 ^aA^
MW-O	3.04 ± 0.86 ^aA^	3.57 ± 0.50 ^aA^	3.91 ± 0.62 ^aA^	3.65 ± 0.52 ^aA^

All values are means ± 95 CI (n = 10). Different lowercase letters in the same row indicate significant differences (*p* < 0.05) based on the biscuit zone. Different capital letters in the same column indicate significant differences for each storage time (*p* < 0.05) based on the baking process.

**Table 6:** QDA of the biscuits as a function of the baking method (O, O-MW and MW-O). Means ± 95 CI (n = 7). Different letters indicate significant differences between samples (*p* = 0.05).

	O	O-MW	MW-O
Color	8.64 ± 0.28 ^a^	6.29 ± 0.70 ^b^	7.50 ± 0.57 ^b^
Toughness	8.00 ± 0.49 ^a^	7.57 ± 0.54 ^a^	9.07 ± 0.54 ^b^
Fragility	4.21 ± 1.26 ^a^	4.43 ± 1.61 ^a^	3.29 ± 1.32 ^a^
Granularity	5.93 ± 0.94 ^a^	5.29 ± 1.24 ^a^	5.86 ± 1.00 ^a^
Mouth residue	5.07 ± 1.56 ^a^	5.07 ± 1.25 ^a^	4.86 ± 1.45 ^a^
Typical flavor	6.71 ± 0.52 ^a^	6.50 ± 0.71 ^a^	6.50 ± 0.43 ^a^
Non typical flavor	0.14 ± 0.28 ^a^	0.00 ± 0.00 ^a^	0.29 ± 0.56 ^a^
Sweetness	4.57 ± 0.40 ^a^	4.57 ± 0.40 ^a^	4.64 ± 0.51 ^a^
Typical odor	6.07 ± 0.94 ^a^	6.29 ± 0.60 ^a^	6.57 ± 0.69 ^a^
Non-typical odor	0.00 ± 0.00 ^a^	0.00 ± 0.00 ^a^	0.00 ± 0.00 ^a^

All values are means ± 95 CI (n = 7). Different letters in the same row indicate significant differences (*p* < 0.05) based on the baking process.

**Table 7:** Energy consumption (EC, Wh/kg of raw biscuits) for each baking process (O, O-MW, MW-O), including both the total and individual phases (O, MW), with and without oven preheating. Total baking time (min) is also provided for comparison.

	O EC (Wh/kg)	MW EC (Wh/kg)	Total EC (Wh/kg)	Total Baking Time (min)
**With oven preheating phase**				
O	831.6 ± 4.9 ^A^	-	831.6 ± 4.9 ^A^	24.5
O-MW	695.2 ± 4.5 ^B^	139.5 ± 2.1 ^A^	834.7 ± 7.5 ^A^	22.2
MW-O	473.4 ± 4.3 ^C^	268.7 ± 3.3 ^B^	742.1 ± 9.2 ^B^	20.5
**Without oven preheating phase**				
O	545.9 ± 2.9 ^A^	-	545.9 ± 2.9 ^A^	16.0
O-MW	409.5 ± 2.2 ^B^	139.5 ± 2.1 ^A^	549.0 ± 5.1 ^A^	13.7
MW-O	187.7 ± 1.0 ^C^	268.7 ± 3.3 ^B^	456.4 ± 5.9 ^C^	12.0

EC values are means ± 95 CI (n = 5). Different capital letters in the same column indicate significant differences for each baking process.

## Data Availability

The original contributions presented in this study are included in the article. Further inquiries can be directed to the corresponding author.
